# Assessment of New and Genome-Reduced *Pseudomonas* Strains Regarding Their Robustness as *Chassis* in Biotechnological Applications

**DOI:** 10.3390/microorganisms11040837

**Published:** 2023-03-25

**Authors:** María José Cárdenas Espinosa, Tabea Schmidgall, Jessica Pohl, Georg Wagner, Benedikt Wynands, Nick Wierckx, Hermann J. Heipieper, Christian Eberlein

**Affiliations:** 1 Department of Environmental Biotechnology, Helmholtz Centre for Environmental Research—UFZ, 04318 Leipzig, Germany; 2 Institute of Bio- and Geosciences, IBG-1: Biotechnology, Forschungszentrum Jülich, 52428 Jülich, Germany

**Keywords:** *Pseudomonas*, biocatalysts, genome-reduced *chassis*, bio-production

## Abstract

Organic olvent-tolerant strains of the Gram-negative bacterial genus *Pseudomonas* are discussed as potential biocatalysts for the biotechnological production of various chemicals. However, many current strains with the highest tolerance are belonging to the species *P. putida* and are classified as biosafety level 2 strains, which makes them uninteresting for the biotechnological industry. Therefore, it is necessary to identify other biosafety level 1 *Pseudomonas* strains with high tolerance towards solvents and other forms of stress, which are suitable for establishing production platforms of biotechnological processes. In order to exploit the native potential of *Pseudomonas* as a microbial cell factory, the biosafety level 1 strain *P. taiwanensis* VLB120 and its genome-reduced *chassis* (GRC) variants as well as the plastic-degrading strain *P. capeferrum* TDA1 were assessed regarding their tolerance towards different *n*-alkanols (1-butanol, 1-hexanol, 1-octanol, 1-decanol). Toxicity of the solvents was investigated by their effects on bacterial growth rates given as the EC50 concentrations. Hereby, both toxicities as well as the adaptive responses of *P. taiwanensis* GRC3 and *P. capeferrum* TDA1 showed EC50 values up to two-fold higher than those previously detected for *P. putida* DOT-T1E (biosafety level 2), one of the best described solvent-tolerant bacteria. Furthermore, in two-phase solvent systems, all the evaluated strains were adapted to 1-decanol as a second organic phase (i.e., OD_560_ was at least 0.5 after 24 h of incubation with 1% (*v/v*) 1-decanol), which shows the potential use of these strains as platforms for the bio-production of a wide variety of chemicals at industrial level.

## 1. Introduction

Biocatalysis is one of the most promising technologies for the sustainable synthesis of complex molecules at biotechnological, pharmaceutical, and industrial scale [1]. The increased demand for bio-based chemicals is driving their growth in the global market, making them economically competitive with fossil raw materials [2]. Particularly with regard to the demand for transforming oil-based chemical production into a sustainable bio-based circular economy, modern techniques of metabolic engineering and synthetic biology have favored the biotechnological production of high-value compounds. However, next to improvements of bioconversion pathways and product yields, the biocatalysts also need to be able to tolerate high, often toxic concentrations of substrates and products, respectively [3]. Often, solvents are used as reservoirs/sinks for the substrates/products. Thus, substrate/product concentrations in the aqueous phase can be kept below inhibitory levels [4]. The reservoir function of the solvent phase represents a big advantage over batch cultures without a second phase, where the bacterial growth conditions can be adverse from the beginning or worsen during the cultivation process. However, the solvents involved in the second phase need to be tolerated by the biocatalysts as well. Biocompatibility of solvents has been widely assessed by log P_o/w_ value, which is the logarithm of the partition coefficient in an octanol–water system [5,6]. Generally, log P_o/w_ in the range of 1 and 4 (e.g., long chain alkanols, aromatics, esters, among others) are toxic to microorganisms at very low concentrations because these solvents accumulate in the cytoplasmic membrane. Partitioning into the membrane lipid bilayer correlates with the compound’s log P_o/w_ and toxicity. Solvents accumulating within the hydrophobic layer of the membrane cause an increase in fluidity [7,8], which leads to inactivation and denaturation of membrane embedded proteins, such as ion pumps and ATPases, it provokes leakage of ions and intracellular macromolecules, such as RNA, phospholipids, and proteins [7]. Among all these effects, the increase in membrane permeability is considered the main reason for cell death [7,8].

Tolerance to hydrocarbon solvents is an evolutionary trait employed by bacterial cells to overcome the stress imposed by these compounds. Tolerance mechanisms have been widely studied in Gram-negative bacteria, especially in various strains of *Pseudomonas* that exhibit an exceptional metabolic versatility and play a relevant role in biotransformation [2,9,10,11]. In the presence of a solvent, several strategies have been observed: (a) adaptive alterations of the membrane fatty acids and phospholipid head group composition, (b) energy-dependent active efflux pumps, and (c) membrane vesicle formation [9,10]. From these mechanisms, changes in phospholipid profile and extrusion of the solvent are considered the most efficient methods of solvent tolerance [12,13]. 

The resistance–nodulation–cell division (RND) efflux pumps are membrane proteins that transport multiple substrates into and out of the cell [14]. The outstanding solvent tolerance of some *Pseudomonas* species can be attributed to efflux pumps directly involved in toluene resistance called TtgABC, TtgDEF, and TtgGHI, whereby the latter was identified as the main pump causing the solvent tolerance phenotype. These pumps confer basal resistance to several solvents including styrene, xylenes, ethylbenzene, and propylbenzene [15]. 

The use of native solvent-tolerant strains has demonstrated several complications in industrial biotechnology. The complications reach from a possible accumulation of toxic intermediates, reduction of biomass formation, imbalances in pathway flux to unpredictable product yields [16]. During the last years, the deletion of dispensable features by metabolic engineering has optimized bioconversion pathways, production levels and strain stability [11,17]. 

Therefore, solvent-tolerant strains of *Pseudomonas* can be seen as perfect biocatalysts for the biotechnological production of various chemicals. However, many strains with the highest tolerance belong to the species *P. putida* that are classified as biosafety level 2 strains in Germany (*P. putida* KT2440 being an exception), which makes them less interesting for the biotechnological industry due to costly but necessary safety measures. This German classification for the *P. putida* group is highly questionable because of the absence of pathogenicity factors from the genome and the history of safe use of many strains. By the U.S. Food and Drug Administration (FDA), the *P. putida* group is considered as non-pathogenic [18]. Moreover, an occasion to review the German classification is the ongoing debate about the *P. putida* group. After a taxonomic review of the *P. putida* clade, a new species of *P. alloputida* was suggested, encompassing well-investigated strains such as *P. putida* KT2440, *P. putida* S12, and *P. putida* DOT-T1E, among others [19]. Next to the aforementioned issues in legislation and taxonomy, it is important to identify new *Pseudomonas* strains’ high tolerance towards solvents and other forms of stress, which are suitable for establishing production platforms of biotechnological processes. In order to exploit the potential of other *Pseudomonas* species for biotechnological applications, solvent-tolerant features were enhanced in *Pseudomonas taiwanensis* VLB120 by successive genome reduction. The deletion of genes related to biofilm formation and flagella expression by the removal of the megaplasmid pSTY and proviral segments provided three new bacterial strains called GRC (genome-reduced *chassis*). In total, the genome was reduced by up to 10%. Strain GRC1 lacks the efflux pump TtgGHI, whereas the *ttgGHI* genes without and with regulatory genes *ttgVW* were re-integrated in GRC2 and GRC3, respectively, enhancing solvent tolerance [20]. Previously, similar genome reductions in *P. putida* KT2440 and in VLB120 considerably enhanced biomass yield coefficients and heterologous gene expression compared to the wildtype [17,20,21]. However, the main disadvantage of KT2440 is its low solvent tolerance when compared to other *Pseudomonas* strains.

Furthermore, the recently isolated strain *P. capeferrum* TDA1 has been reported as a plastic monomer and oligomer degrader [22,23], which shows its metabolic capacity and broad potential to degrade recalcitrant compounds. Together with three *P. putida* KT2440 derivatives in a defined microbial mixed culture, TDA1 was deployed in the metabolization of polyurethane hydrolysates. The mineralization of the polyurethane hydrolysates led to the subsequent production of rhamnolipids [24]. The study reflects the important role that new bacterial strains can play in the (bio)technological plastic upcycling.

In this study, the different solvent tolerance levels towards *n*-alkanols (1-butanol, 1-hexanol, 1-octanol, and 1-decanol) in *Pseudomonas taiwanensis* VLB120, GRC1, GRC2, GRC3, and in *Pseudomonas capeferrum* TDA1, and their performance in two-phase systems as potential hosts for the bio-production of chemical compounds at industrial level is described. The *n*-alkanols with different log P_o/w_ values (0.89 for 1-butanol, 1.87 for 1-hexanol, 2.92 for 1-octanol, and 3.97 for 1-decanol) function as benchmarks to compare the solvent tolerance and their suitability for biotechnology, respectively, of genome-reduced *chassis* strains compared to the wildtype *P. taiwanensis* VLB120.

## 2. Materials and Methods

### 2.1. Strains

Strain *Pseudomonas taiwanensis* VLB120 and its genome-reduced strains GRC1, GRC2, and GRC3 were selected for the stress assessment because of their superior bioprocess features compared to the wildtype [20]. The genome for the three strains was reduced by about 10%. The GRC strains lack genes enabling the cells to swim and form biofilms. Moreover, the megaplasmid pSTY and large proviral segments were deleted from the genome. For *Pseudomonas taiwanensis* GRC2 and GRC3 the genes for the efflux pump TtgGHI (formerly present on the deleted megaplasmid pSTY) were reintroduced. For *Pseudomonas taiwanensis,* GRC3 as well the regulatory genes *ttgVW* were reintegrated into the bacterial chromosome (Table 1). 

Additionally, the bacterial strain *Pseudomonas capeferrum* TDA1 was included in the assessment, based on reports about its metabolic ability to degrade a polyurethane monomer and a polyurethane oligomer [22] and first applications in biotechnological approaches [24]. The genome of *Pseudomonas capeferrum* TDA1 was first published under the species name *Pseudomonas* sp. TDA1. An improved genome assembly for *Pseudomonas capeferrum* TDA1 is now available under the accession number CP116669.1.

### 2.2. Medium and Culture Conditions

All bacterial strains were regularly grown in “Hartmans” mineral medium [25] and Na_2_-succinate (4 g/L) as a carbon and energy source at 30 °C and 180 rpm (overnight culture). A fresh culture was inoculated with an overnight culture to reach an OD_560_ of about 0.1. The OD of the new culture was measured every hour and during the exponential phase, the *n*-alkanols (1-butanol, 1-hexanol, 1-octanol, and 1-decanol) were added at different concentrations, separately, according to their log P_o/w_ value. 1-Butanol was added in the range of 10 to 400 mM, 1-hexanol from 1 to 15 mM, 1-octanol from 0.1 to 2.5 mM, and 1-decanol from 0.05 to 0.4 mM. Purchased solutions of 1-octanol and 1-decanol were diluted with acetone to facilitate the addition to the cultures and the solubility in the mineral medium. Before reaching the stationary phase, the cultures were centrifuged (15 min at 10,000 rpm) (Heraeus, Hanau, Germany) and the pellets were resuspended in 1.75 mL of phosphate buffer (50 mM at pH 7.0). Finally, the bacterial cells were centrifuged (7 min at 13,000 rpm) and stored at −20 °C. Growth inhibition (Formula (2)) caused by the toxic compounds was measured by comparing the percentage difference in the growth rates µ (h^−1^) (Formula (1)) between intoxicated cultures with that of control [26]. 

Formula (1): Calculation of bacterial growth rates µ (h^−1^)

(1) $$\mathrm{Growth}\;\mathrm{rate}\;µ\;\left(h^{-1}\right)=\frac{{\mathrm{Ln}\;\mathrm{OD}}_{t1}-{\mathrm{Ln}\;\mathrm{OD}}_{t0}}{t_{1}-t_{0}}$$

Formula (2): Calculation of growth inhibition in %

(2) $$\mathrm{Inhibited}\;\mathrm{growth}\;\left(\%\right)=\frac{{µ}_{\mathrm{with}\_\mathrm{toxin}}\times 100}{{µ}_{\mathrm{control}}}$$

### 2.3. Extraction of Membrane Lipids

The resulting pellets, equivalent to a dry mass of about 15 mg, were suspended in 0.5 mL of water, 1 mL of methanol and 1.75 mL of chloroform. The solution was shaken for 3 min using a vortex (VWR, West Chester, PA, USA), and 0.5 mL of water was added to the mixture, which was agitated for 30 s. Then, the solution was centrifuged (10 min at 3000 rpm) and the chloroform phase was transferred to HPLC-flasks. 

For the methylation of fatty acids, samples were incubated in BF_3_-methanol (Merck, Darmstadt, Germany) for 15 min at 95 °C by applying the method of Morrison and Smith [27]. Lastly, fatty acid methyl esters (FAME) were extracted with hexane and stored at 4 °C. 

### 2.4. Determination of Fatty Acid Composition

FAME analysis was performed using gas chromatography with flame ionization detector (GC-FID, Agilent Technologies, 6890N Network GC System, 7683B Series Injector). The instrument used a CP-Sil 88 column (Varian CP7488) in stationary phase and helium as a carrier gas. The temperature program was 40 °C, 2 min isothermal, a gradient increase up to 220 °C (8 °C × min^−1^), and 10 min at 220 °C. The peak areas of the FAMEs were used to determine their relative amounts. The fatty acids were identified by co-injection of authentic reference compounds obtained from Supelco (Bellefonte, PA). *Trans/cis* ratio was calculated taking the sum of the FAME of palmitoleic acid (C16:1Δ9*cis*) and *cis*-vaccenic acid (C18:1Δ11*cis*) as divisor and the sum of their corresponding *trans* configuration as dividend [7].

### 2.5. Growth in in a Second Phase System of 1-octanol and 1-decanol

All bacterial strains were cultivated in mineral medium and Na_2_-succinate as described above for 5 h. After reaching an OD_560_ of 0.5, 1 mM (0.015% *v*/*v*) of 1-octanol or 0.2 mM (0.004% *v*/*v*) of 1-decanol were added to different assays, separately. The cultures grew for 3 h and then, 10 mL of these cultures were transferred to a fresh medium to reach an OD_560_ of about 0.1. Subsequently, 1% (*v*/*v*) of 1-octanol (maximum concentration in the aqueous phase of 3.8 mM) or 1-decanol (maximum concentration in the aqueous phase of 0.23 mM) were added to the corresponding assays. These cultures grew overnight and the OD_560_ was measured after 24 h for all strains. The tested strains were considered as *adapted* at an OD_560_ of at least 0.5 or *well-adapted* at an OD_560_ of over 1 after 24 h incubation with 1% (*v*/*v*) of 1-octanol or 1-decanol. 

### 2.6. Statistical Analysis

Data of at least threefold measurements were obtained as the mean ± one standard deviation. Student’s *t*-test was used to analyze the significant differences (*p* < 0.05).

## 3. Results and Discussion

### 3.1. Growth Kinetics 

**Figure 1:**
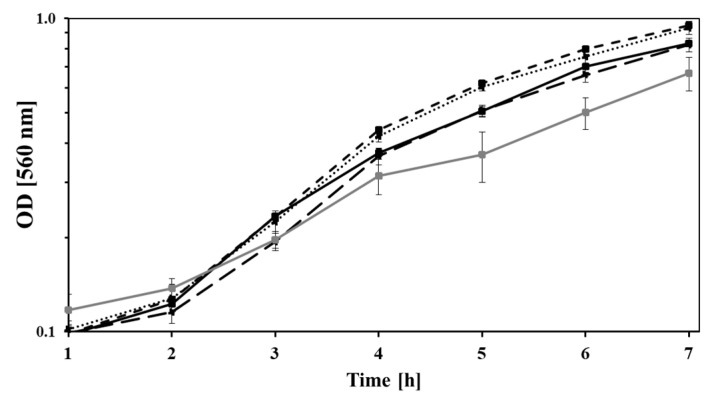
OD_560_ during the growth of *P. taiwanensis* VLB120 (solid black line), *P. taiwanensis* GRC1 (dotted black line), GRC2 (long dashed black line), GRC3 (short dashed black line), and *P. capeferrum* TDA1 (solid gray line) on Na_2_-succinate (4 g/L). Error bars indicate the standard error of the mean (n = 3).

In order to assess the impact of the genome reduction on the growth kinetics, all the aforementioned strains were cultivated in mineral medium with Na_2_-succinate as carbon and energy source. The growth rate of *P. taiwanensis* GRC3 (µ = 0.38 ± 0.02 h^−1^) and GRC1 (µ = 0.37 ± 0.01 h^−1^) increased up to 5.2% compared to *P. taiwanensis* VLB120 (µ= 0.36 ± 0.01 h^−1^) (Figure 1). Growth rates of GRC2 (µ = 0.35 ± 0.015 h^−1^) and TDA1 (µ = 0.29 ± 0.04 h^−1^) were slightly below the rates of the aforementioned strains.

### 3.2. Effects of n-alkanols on Bacterial Growth 

**Figure 2:**
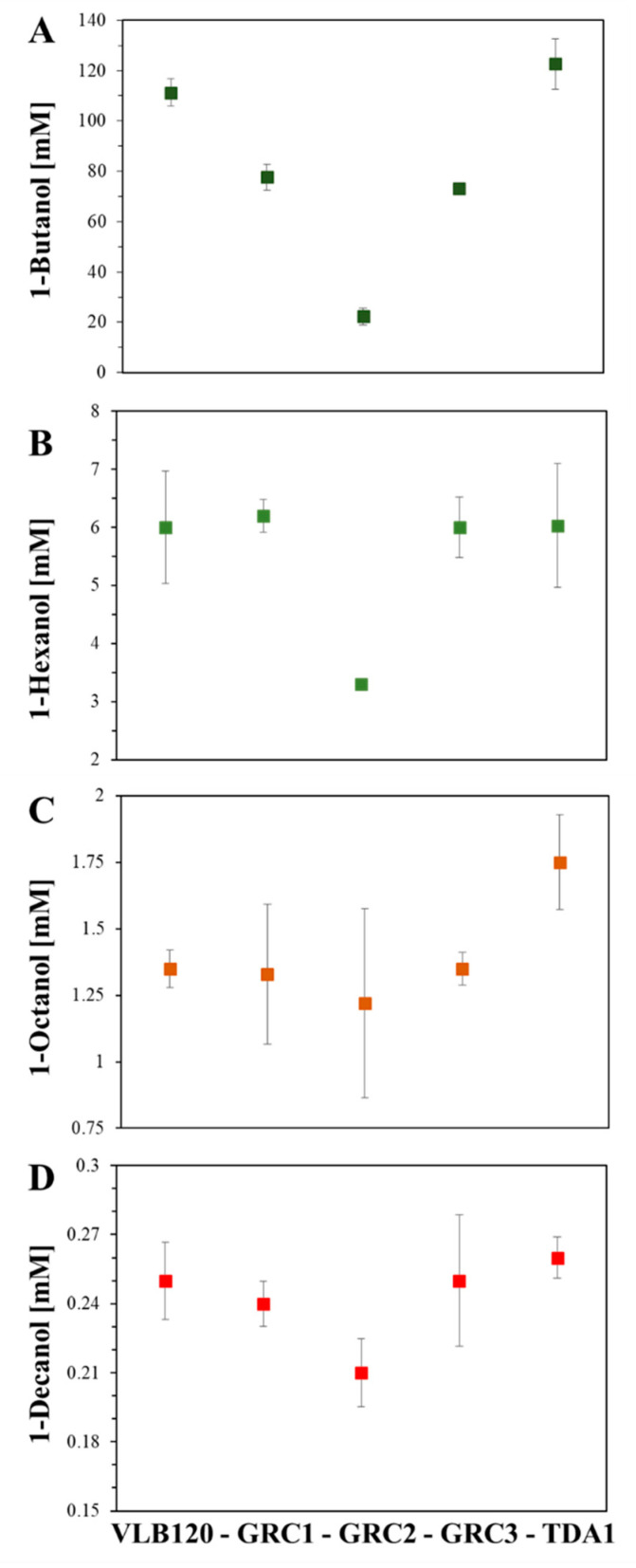
EC50 values for *P. taiwanensis* VLB120, GRC1, GRC2, GRC3, and *P. capeferrum* TDA1 grown on (**A**) 1-butanol (dark green squares), (**B**) 1-hexanol (green squares), (**C**) 1-octanol (orange squares), and (**D**) 1-decanol (red squares).

The effect of *n*-alkanols (1-butanol, 1-hexanol, 1-octanol, and 1-decanol) of different log P_o/w_ on the growth of *P. taiwanensis* VLB120 (wildtype), GRC1, GRC2, GRC3, and *P. capeferrum* TDA1 was assessed using EC50 (effective concentration for reducing cell growth by 50% compared to the control) [28]. Previous reports have shown that higher hydrophobicity leads to a higher tendency to accumulation in the membranes [4,29]. The most important finding of this study is that the GRC strains not only show better bioprocess features compared to the wildtype VLB120, as reported before [20], but that their tolerance towards the tested *n*-alkanols is also comparable. Thus, the genome reduction process did not diminish solvent tolerance towards *n*-alkanols (Figure 2 and Table 2). It is likely that this counts also for the tolerance towards other solvents such as toluene and styrene in GRC2 and GRC3 because of the presence of the TtgGHI pump.

Some Pseudomonads harbor several RND-type efflux pumps (TtgABC, TtgDEF, and TtgGHI) that are involved in the extrusion of numerous solvents. For instance, TtgABC mainly extrudes flavonoids, antibiotics, and short-chain alcohols such as *n*-butanol [30,31]. TtgGHI efflux pump seems to be induced only by aromatic hydrocarbons such as toluene, whereas TtgDEF plays an important role in tolerance to aliphatic alcohols [32] and organic solvents such as toluene, ethylbenzene, propylbenzene, and styrene [11]. The targeted elimination of the pSTY megaplasmid in tailored strain GRC1, re-integration of the pSTY-encoded efflux pump genes *ttgGHI* without (GRC2) and with (GRC3) transcriptional regulators enhanced solvent tolerance and performance indicators such as titer, yield, and product tolerance [20,33].

Previously, the TtgABC efflux pump was actively expressed in *Escherichia coli* at high concentrations of short-chain alcohols (*n*-butanol, isobutanol, isoprenol, and isopentanol) inhibiting bacterial growth. Under these conditions, the function of TtgABC was limited to improving survival, rather than supporting bacterial growth [30]. Solvent tolerance mechanisms are considered energy-intensive processes that come at the cost of growth yields to protect cells from further damage [4,34]. In the presence of sub-lethal toluene dosages, *P. putida* S12 showed a marked decrease in growth yield, which was reduced linearly with increasing toluene concentrations [35]. It is likely that the constitutive expression of the TtgGHI efflux pump in *P. taiwanensis* GRC2 has required high levels of energy and, in addition, the presence of 1-butanol and 1-hexanol might have induced other RND-type efflux systems [36], which could also increase the energy consumption, significantly reducing bacterial growth. These results showed a competitive advantage of GRC1 and GRC3 compared to GRC2 enhancing key performance indicators that can be applicable to many biotechnological processes. On the other hand, *P. taiwanensis* GRC2 showed similar EC50 values in assays containing 1-octanol and 1-decanol (1.22 ± 0.35 and 0.21 ± 0.01 mM, respectively) compared to *P. taiwanensis* VLB120 (1.35 ± 0.07 and 0.25 ± 0.01 mM), *P. taiwanensis* GRC1 (1.33 ± 0.26 and 0.24 ± 0.00 mM), and GRC3 (1.35 ± 0.06 and 0.25 ± 0.02 mM), respectively. Comparable results were observed in the tested wildtypes, GRC1 and GRC3, where the EC50 values were similar for all the strains in 1-butanol, 1-hexanol, 1-octanol, and 1-decanol (Figure 2). However, GRC1 was previously described as a sensitive strain in the presence of hydrophobic solvents such as toluene [20] which points to a preference for GRC2 and GRC3 for corresponding applications in microbial biocatalysis. In cases where aromatics are gradually accumulating such as in de novo production [31], *P. taiwanensis* GRC2 or GRC3 might provide a balance between optimal growth and solvent tolerance in high-stress conditions due to the presence of the *ttgGHI* genes. In addition, as almost exclusively typical for *Pseudomonas*, an increase in the *trans/cis* ratio, dependent on the stressor concentration, was observed (data for GRC3 shown in Figure S1). This shows the functionality of this fast stress response mechanism also in the genome-reduced *chassis* strains of *P. taiwanensis* [9,28].

*P. capeferrum* TDA1 showed the highest EC50 values for all the assays (1-butanol, 1-hexanol, 1-octanol, and 1-decanol) (122.66, 6.03, 1.75, and 0.26 mM, respectively). The strain TDA1 contains the *ttgABC* genes; however, none of the gene sets for TtgGHI nor for TtgDEF are complete. Several genes related to biofilm formation and induction, multidrug resistance proteins and RND transporters were identified as differentially expressed (upregulated) in *P. capeferrum* TDA1 grown on the aromatic compound 2,4-diaminotoluene [22], however, *ttgABC* were not among them. Thus, TtgABC was most probably not involved in the adaptation to 2,4-diaminotoluene. According to the EC50 values obtained in the present study, TtgABC could be rather involved in the adaptation to *n*-alkanols. However, this remains an open question for further studies. In Rojas et al. 2004, a *P. putida* DOT-T1E mutant lacking *ttgH* did not show lower tolerance to 1-octanol, 1-nonanol, and 1-decanol [32]. This is in full agreement with the data shown in Figure 2, where GRC1 is equally tolerant to 1-octanol and 1-decanol as the VLB120 wildtype. Furthermore, it was suggested by Rojas et al. 2004 that the aliphatic alcohols’ extrusion was performed simultaneously by the three efflux pumps (TtgABC, TtgDEF, and TtgGHI) present in DOT-T1E because a triple mutant lacking all the pumps was much more sensitive to the *n*-alkanols tested [32]. However, further evidence is needed regarding that. Additionally, efflux pumps in *P. taiwanensis* VLB120 may have different substrate ranges than those in *P. putida* DOT-T1E. Constitutive expression of *ttgGHI* gave a clear fitness advantage to *P. taiwanensis* GRC2 compared to GRC1 or GRC3 when confronted with hydrophobic aromatics such as styrene, toluene, or 4-ethylphenol [20]. However, GRC2 showed a marked growth defect when confronted with the more hydrophilic aromatic phenol. Together, this strongly suggests that the TtgGHI pump is probably specific for hydrophobic aromatic substrates, and only poses an energetic burden for GRC2 when facing *n*-alkanols. The adaptive response of *P. taiwanensis* GRC3 and *P. capeferrum* TDA1 revealed a superior performance with EC50 values up to two-fold higher than those presented in *P. putida* DOT-T1E formerly [4] (Table 2). These results revealed the potential use of these strains as platforms for the bio-production of a wide variety of chemicals at industrial level. 

### 3.3. Two-Phase Adaptation of 1-octanol and 1-decanol in P. taiwanensis VLB120, GRC1, GRC2, GR3, and P. capeferrum TDA1

Solvent-tolerant bacteria are well suited for biocatalytic production in two-phase systems [2]. One major problem—next to the substrate and product toxicity—concerns product recovery from the aqueous medium. A promising solution addressing both problems is the implementation of a two-phase system with an organic solvent as the second liquid phase. Since the product accumulates in the organic solvent phase, it can be isolated by routine chemical purification methods [4,10,37]. Nevertheless, due to the toxic effects, only few solvents with high hydrophobicity coefficients can be applied for this purpose, which limit their use at industrial applications involving microbial activity. To enhance tolerance to chemical stresses in bacteria, it is needed to increase yields and titers of several bioprocesses [38]. 

Organic solvents with log P_o/w_ ≥ 2.5 (1-octanol, 1-nonanol, and 1-decanol) were previously tested to predict the adaptation of *Pseudomonas putida* DOT-T1E in a second-phase system. The results revealed that pre-exposed bacterial cells to the aforementioned solvents were able to adapt, whereas an increased amount of unsaturated *trans*- and saturated membrane fatty acids was crucial [4,32]. In this study, the growth of *P. taiwanensis* VLB120, GRC1, GRC2, GRC3, and *P. capeferrum* TDA1 was evaluated in a two-phase system with 1% (*v*/*v*) of 1-octanol or 1-decanol (Table 3). The cultures were preadapted with 1 mM of 1-octanol or 0.2 mM of 1-decanol, respectively, and subsequently cultivated in medium containing 1% (*v*/*v*) of 1-octanol or 1-decanol for 24 h. The tested strains were considered as adapted at an OD_560_ of at least 0.5 or well-adapted at an OD_560_ of over 1 after 24 h incubation with 1% [*v*/*v*] of 1-octanol or 1-decanol. *P. taiwanensis* VLB120 and *P. capeferrum* TDA1 adapted to these solvents in a second-phase system (OD_560_ 0.73 ± 0.02 and 0.71 ± 0.18, respectively). However, the streamlined GRC strains showed a faster adaptability (OD_560_ above 1). This can be explained most probably due to their higher growth rates compared to the wildtype strain tested. 

In contrast to 1-octanol, all the tested strains were able to adapt very well to 1-decanol. One parameter to predict the toxicity of several compounds is the maximum membrane concentration (MMC) [39]. For long-chain *n*-alkanols such as 1-octanol and 1-decanol, the MMC is 588 mM and 379 mM, respectively. Values higher than 200 mM are considered extremely toxic to microorganisms [40]. The lower MMC of 1-decanol explains the better adaptability to it in contrast to 1-octanol. This was documented for *P. putida* DOT-T1E and *P. putida* KT2440 before [4,41].

The addition of 1-decanol as a second phase has been widely used to alleviate product toxicity and increase productivity and product titers at industrial level [42]. Moreover, the inherent solvent tolerance of *Pseudomonas* is considered an important trait for the bio-production of chemicals. For instance, *P. putida* S12 has been used for the production of *p*-hydroxystyrene in a second phase of 1-decanol, which resulted in a four-fold increased titer compared to a standard fed-batch production [42]. *P. putida* S12 and an engineered *E.coli* TG1-326 expressing the solvent efflux pump SrpABC were employed as hosts for the production of 1-naphthol in a water-organic solvent (1-decanol) biphasic system [43]. *Pseudomonas* enhanced 1-naphthol production by approximately 42% contrasted with *E. coli* under the same conditions, which demonstrated that *Pseudomonas* is a more robust candidate for whole-cell bioprocesses.

## 4. Conclusions and Future Perspectives

The transition of the current fossil-based to a bio-based economy requires specialized microbial cell factories for the production of industrially relevant compounds. Despite this ongoing progress, the number of biotechnologically produced compounds at commercial scale is still very limited. The unique solvent-tolerant features of *P. taiwanensis* VLB120 in combination with tailored metabolic modifications provides several advantages including genetic stability and enhanced fitness under stress-triggering conditions. This study could show that the genome-reduced strains of *P. taiwanensis* VLB120 are in no way inferior to the wildtype in terms of tolerance. Therefore, the streamlined *P. taiwanensis* GRC3 with the TtgGHI pump to be activated on demand has all the properties required by the industry to be used as a new chassis bacterium, such as superior solvent tolerance to solvents even in two-phase systems. With the recent advances in high-throughput genome editing and evolution, more strains can be considered as attractive new host platforms, reducing host interference and increasing production yields. The potential of streamlined *chassis* strains for whole-cell biocatalytic applications might improve the development of new bio-based chemicals replacing petrochemical-based production in the near future. 

## Figures and Tables

**Table 1:** Strains used in the study, their abbreviation, and the presence (+) or absence (−) of the full set of genes for the efflux pump TtgGHI and its regulators TtgVW. GRC: genome-reduced chassis, TDA: toluene diamine.

Strain	Abbreviation	*ttgTGH*	*ttgVW*
***P. taiwanensis*** **VLB120**	VLB120 or wildtype	+	+
***P. taiwanensis*** **VLB120 GRC1**	GRC1	−	−
***P.taiwanensis*** **VLB120 GRC2**	GRC2	+	−
***P.taiwanensis*** **VLB120 (GRC3)**	GRC3	+	+
***P. capeferrum*** **TDA1**	TDA1	−	−

**Table 2:** EC50 values (mM) of the tested *n*-alkanols in *P*. *putida* DOT-T1E (data from [4]), *P. taiwanensis* GRC3 (see also Figure S1), and *P. capeferrum* TDA1.

*n*-alkanols	*P*. *putida* DOT-T1E [4]	*P. taiwanensis* GRC3	*P. capeferrum* TDA1
**1-Butanol**	49	73	123
**1-Hexanol**	6.35	6	6
**1-Octanol**	0.80	1.38	1.75
**1-Decanol**	0.11	0.25	0.26

**Table 3:** Bacterial growth (OD_560_) adaptation to a second phase of 1-octanol and 1-decanol present in concentrations of 1% (*v*/*v*). +: adapted (OD_560_ between 0.5 and 1 after 24 h incubation with 1% (*v*/*v*)), ++: well-adapted (OD_560_ above 1 after 24 h incubation with 1% (*v*/*v*)). The errors are given as standard error of the mean (n = 3).

Strain	1-Octanol OD	1-Octanol Adaptation	1-Decanol OD	1-Decanol Adaptation
***P. taiwanensis*** **VLB120**	0.73 ± 0.02	+	1.25 ± 0.31	++
***P. taiwanensis*** **GRC1**	1.33 ± 0.09	++	1.36 ± 0.14	++
***P. taiwanensis*** **GRC2**	1.25 ± 0.16	++	1.47 ± 0.21	++
***P. taiwanensis*** **GRC3**	1.07 ± 0.11	++	1.72 ± 0.34	++
***P. capeferrum*** **TDA1**	0.71 ± 0.18	+	2.28 ± 0.10	++

## Data Availability

The genome assembly for *Pseudomonas capeferrum* TDA1 is available under the accession number CP116669.1. The data presented in this study are available in Figure S1 (Supplemental Material) and on request from the corresponding author.

## Supplementary Materials

The following supporting information can be downloaded at: https://www.mdpi.com/article/10.3390/microorganisms11040837/s1, Figure S1. Growth rates (black circles) and *trans*/*cis* ratios (white diamonds) of *P. taiwanensis* GRC3 incubated with different *n*-alkanols: (A) 1-butanol, (B) 1-hexanol, (C) 1-octanol and (D) 1-decanol.
