# Supplementary material for: Assessment of New and Genome-Reduced Pseudomonas Strains Regarding Their Robustness as Chassis in Biotechnological Applications
Source: Microorganisms. 2023 Mar 25;11(4):837. doi: 10.3390/microorganisms11040837 (PMC10144732; doi:10.3390/microorganisms11040837)
Supplement: Supplementary file 1 [file microorganisms-11-00837-s001.zip › microorganisms-2241513-supplementary.pdf]

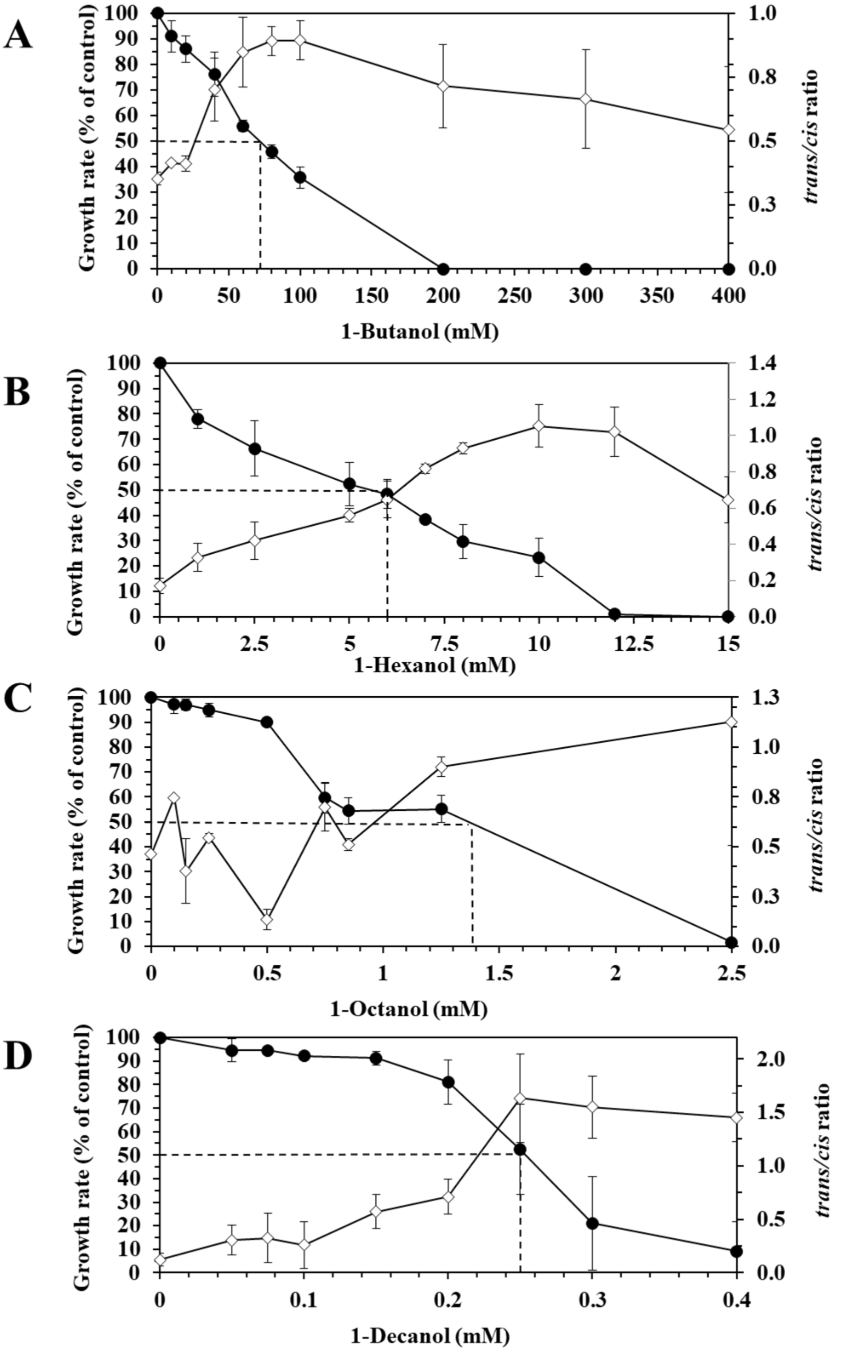

Figure S1. Growth rates (black circles) and *trans*/*cis* ratios (white diamonds) of *P. taiwanensis* GRC3 incubated with different *n*-alkanols: (A) 1-butanol, ()B) 1-hexanol, (C) 1-octanol and (D) 1-decanol.
